# Supplementary material for: Spin-polarized imaging of the antiferromagnetic structure and field-tunable bound states in kagome magnet FeSn
Source: Sci Rep. 2022 Aug 25;12:14525. doi: 10.1038/s41598-022-18678-8 (PMC9411549; doi:10.1038/s41598-022-18678-8)
Supplement: Supplementary file 1 — Supplementary Information. [file 41598_2022_18678_MOESM1_ESM.pdf]

Spin-polarized imaging of the antiferromagnetic structure and field-tunable bound states in kagome magnet FeSn

Hong Li[1], He Zhao[1], Qiangwei Yin[2], Qi Wang[2], Zheng Ren[1], Shrinkhala Sharma[1], Hechang Lei[2], Ziqiang Wang[1], and Ilija Zeljkovic[1]

[1]Department of Physics, Boston College, Chestnut Hill, MA 02467
[2]Department of Physics and Beijing Key Laboratory of Opto-electronic Functional Material & Micro-nano Devices, Renmin University of China, Beijing 100872, China

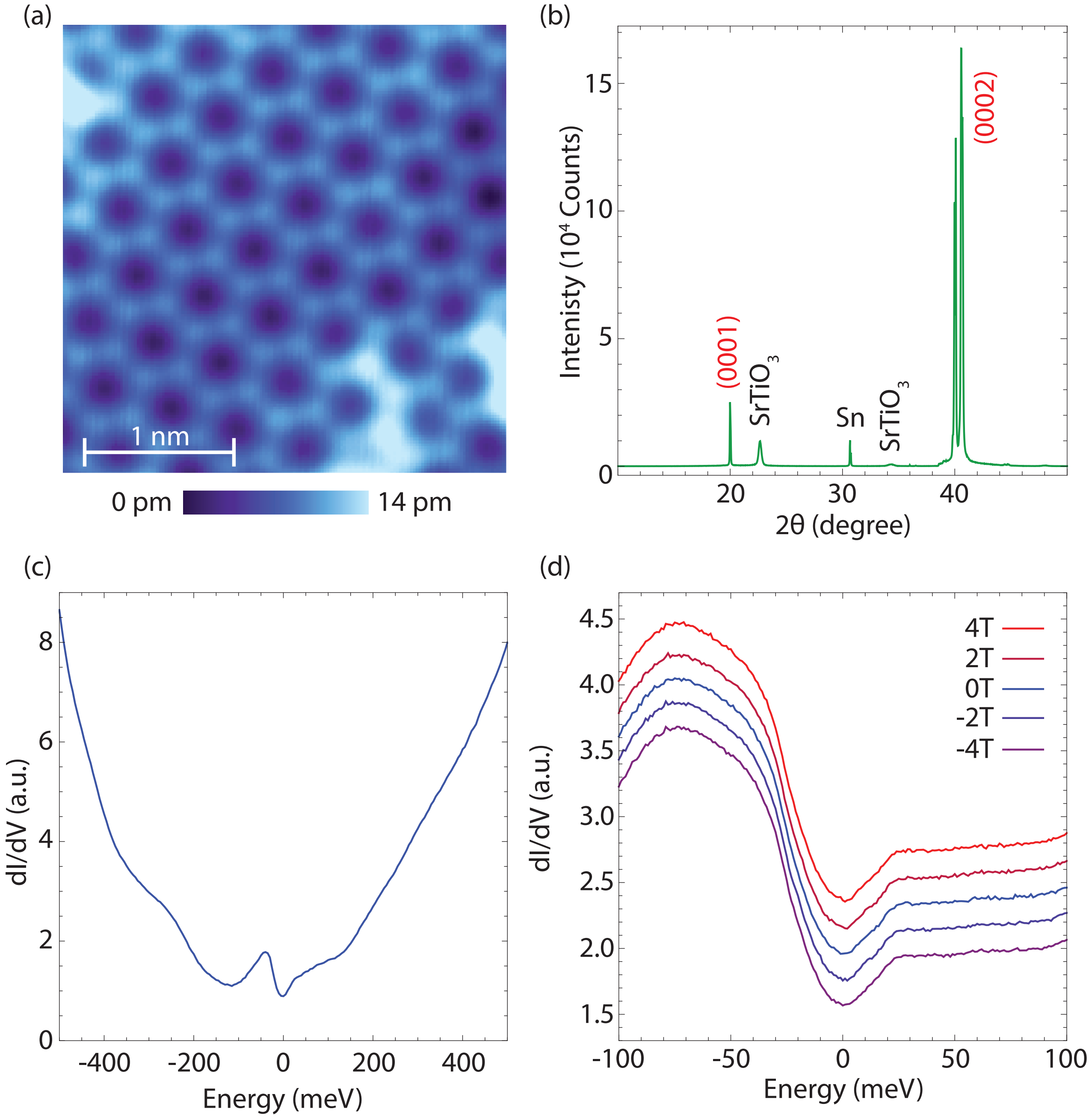

Figure S1: STM/S of home-grown thin film of FeSn. **(a)** STM topograph of the Sn termination of an FeSn thin film grown by MBE, transferred to STM in UHV using a vacuum suitcase. **(b)** Post-growth XRD diffraction pattern of the thin film demonstrating the crystal structure. **(c)** Average dI/dV spectrum taken over the region in (a) at 0 T field. The -50 meV hump is clearly seen, similar to what we find on the Sn surface of bulk FeSn. **(d)** dI/dV spectra taken on region (a) in magnetic fields from -4 T to 4 T, which appear indistinguishable. This it is the same as we observed on the bulk samples, where the peak does not shift with magnetic field using a spin-averaged STM tip. STM setup conditions: (a) I_{set} = 200 pA, V_{sample} = 100 mV; (c) I_{set} = 500 pA, V_{sample} = 500 mV, V_{exc} = 5 mV; (d) I_{set} = 200 pA, V_{sample} = 100 mV, V_{exc} = 1 mV.

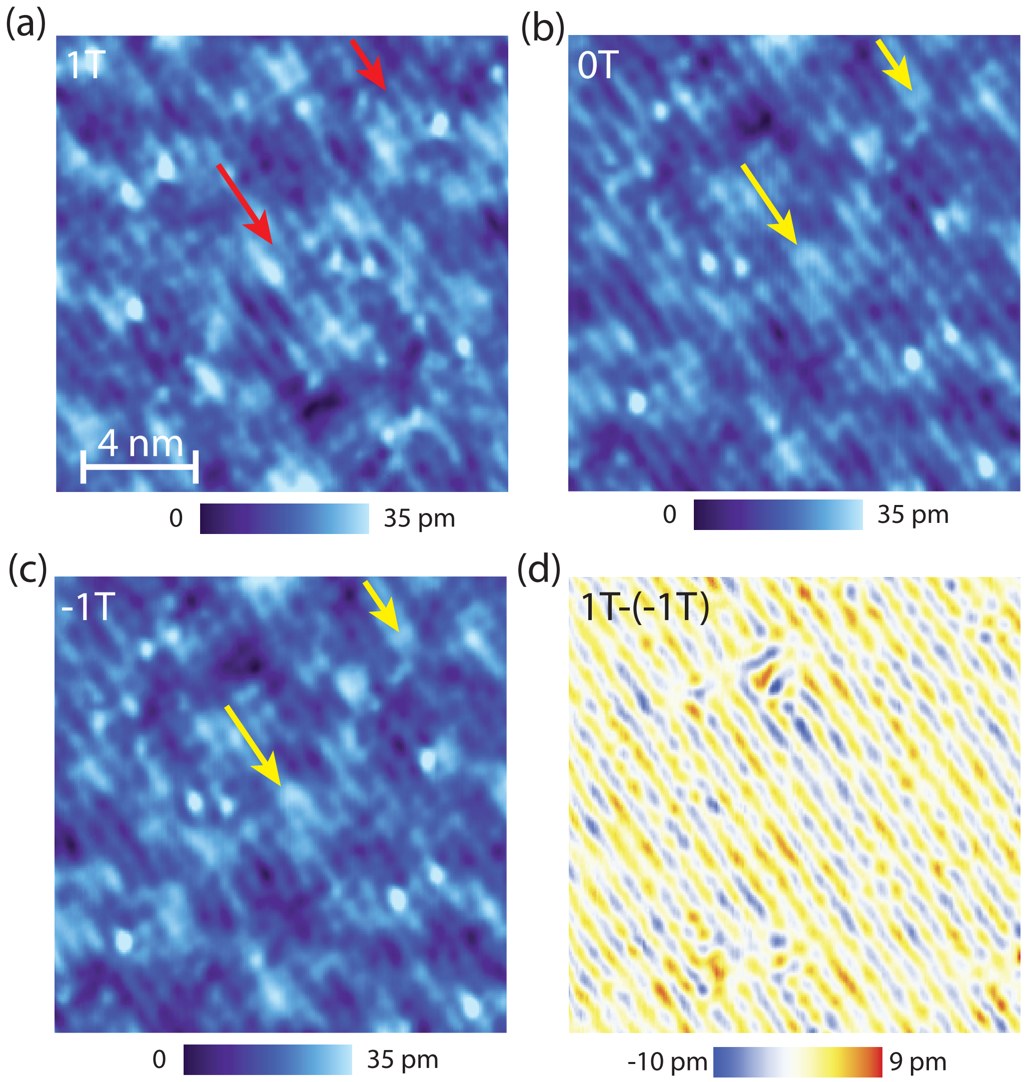

Figure S2: Spin-polarized STM tip characterization on a UHV-cleaved bulk single crystal of FeTe. **(a-c)** STM topographs of FeTe scanned with the same tip in different magnetic fields, showing the well-known $2a_0$ modulations due to the in-plane antiferromagnetic structure of FeTe. Topographs taken on the same FeTe surface region in 1 T and -1 T field show a dark-bright stripe shift (note the difference in features outlined by yellow vs red arrows). This demonstrates that small fields can be used to "flip" the polarization direction of the STM tip. **(d)** Difference between topograph taken at $+1$ T and -1 T, which shows the clear $2a_0$ modulation more obviously. STM setup conditions: (a,b,c) $I_{set} = 600$ pA, $V_{sample} = 100$ mV.

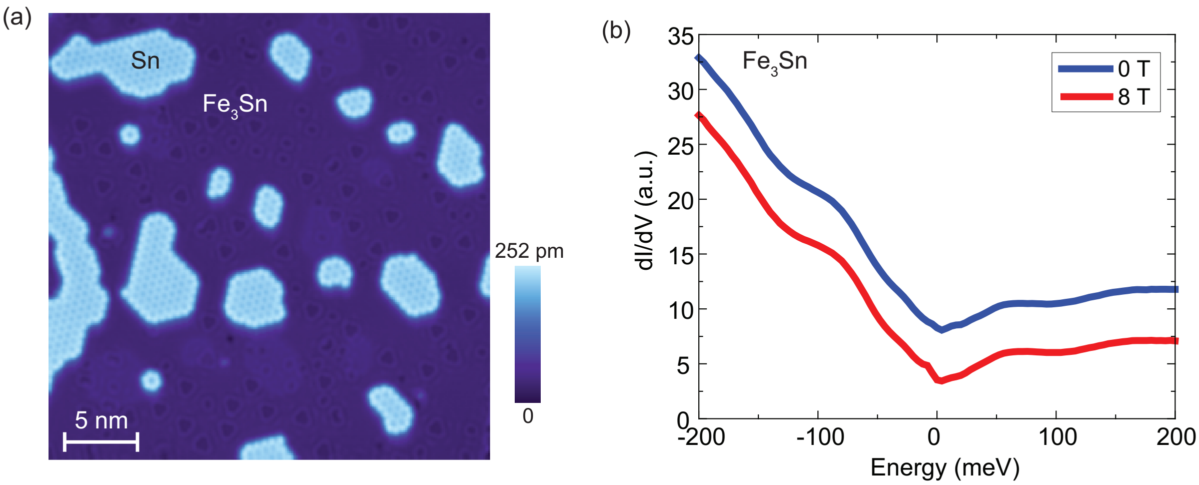

Figure S3: Field-independent spectra on the kagome termination. **(a)** STM topograph of the Fe_3Sn kagome surface with tiny hexagonal Sn islands on top. **(b)** Average dI/dV spectra on the Fe_3Sn surface at 0 T (blue) and 8 T (red). 0 T spectrum is vertically offset by 5 a. u. for clarity. STM setup condition: (a) I_{set} = 800 pA, V_{sample} = 200mV; (b) I_{set} = 800 pA, V_{sample} = 200 mV, V_{exc} = 2 mV.
